# Distribution and Transmission of Colistin Resistance Genes *mcr-1* and *mcr-3* among Nontyphoidal Salmonella Isolates in China from 2011 to 2020

**DOI:** 10.1128/spectrum.03833-22

**Published:** 2023-01-10

**Authors:** Tingting Yang, Weiwei Li, Qingpo Cui, Xiaoxia Qin, Bosheng Li, Xiugui Li, Huayun Jia, Xiaorong Yang, Chengwei Liu, Yang Wang, Shaolin Wang, Jianzhong Shen, Yunchang Guo, Zhangqi Shen

**Affiliations:** a Beijing Key Laboratory of Detection Technology for Animal-Derived Food Safety, College of Veterinary Medicine, China Agricultural University, Beijing, China; b Guangdong Laboratory for Lingnan Modern Agriculture, Guangzhou, China; c National Health Commission Key Laboratory of Food Safety Risk Assessment, Chinese Academy of Medical Science Research Unit (No. 2019RU014), China National Center for Food Safety Risk Assessment, Beijing, China; d Guangdong Provincial Center for Disease Control and Prevention, Guangzhou, China; e Guangxi Provincial Center for Disease Control and Prevention, Nanning, China; f Hunan Provincial Center for Disease Control and Prevention, Changsha, China; g Sichuan Provincial Center for Disease Control and Prevention, Chengdu, China; h Jiangxi Provincial Center for Disease Control and Prevention, Jiangxi, China; i Department of Preventive Veterinary Medicine, College of Veterinary Medicine, China Agricultural University, Beijing, China; Department of Clinical Laboratory, Peking University People’s Hospital, Beijing, China

**Keywords:** nontyphoidal *Salmonella*, *mcr*, colistin, plasmid, genome

## Abstract

Mobile colistin resistance (*mcr*) genes are present mainly in plasmids and can disseminate clonally or horizontally via either plasmids or insertion sequences in different genomic locations among the *Enterobacteriaceae*. A nationwide large-scale study on *mcr* prevalence and transmission in nontyphoidal Salmonella isolates is still lacking. Here, we identified 140 *mcr*-positive Salmonella isolates out of 7,106 isolates from 29 provinces in China from 2011 to 2020. We aligned short reads to putative plasmids from long-read hybrid assemblies and predicted the plasmid backbones of non-long-read sequencing isolates to elucidate *mcr* transmission patterns. The *mcr-1* and *mcr-3* genes are transmitted on similar high-risk clones (sequence type 34 [ST34]) but through plasmids of various replicon types. Furthermore, the ban on colistin use in food animals can lead to a decrease in the *mcr*-positive Salmonella prevalence among diarrheal patients, related mainly to IncHI2A_IncHI2 plasmids. We provide a framework for plasmid data incorporation into genomic surveillance systems, contributing to a better understanding of *mcr* spread and transmission.

**IMPORTANCE** Nontyphoidal Salmonella is one of four major causative agents of diarrheal diseases globally, with most cases of salmonellosis being mild. Antimicrobial treatments are required for cases of life-threatening infections, and colistin is one of the last-line antibiotics for the treatment of multidrug-resistant Salmonella infections. However, the efficacy of colistin has been compromised by the emergence of various *mcr* genes. To elucidate the transmission of *mcr* genes in Salmonella isolates, our study analyzed 7,106 Salmonella strains from 29 provinces in China from 2011 to 2020. The results showed that *mcr* genes are transmitted on similar high-risk clones (ST34) but through plasmids of various replicon types. In addition, our data illustrated that the ban on the use of colistin in food animals led to a significant decrease in *mcr*-positive isolates. Our findings offer an essential step toward a more comprehensive understanding of the spread and transmission of *mcr* genes.

## INTRODUCTION

Antimicrobial resistance (AMR), a major threat to public health, was associated with 4.95 million deaths in 2019 alone, according to a recent global systematic analysis (1). In 2017, the World Health Organization (WHO) announced 12 drug-resistant bacteria that pose the greatest threat to human health and for which new antibiotics are desperately needed, including carbapenem-resistant *Enterobacteriaceae* (CRE) and fluoroquinolone-resistant Salmonella (2). Colistin, defined as a critically important antimicrobial for human health by the WHO in 2018 (3), is one of the last-line antibiotic classes for the treatment of multidrug-resistant (MDR) pathogens (4, 5). However, colistin’s efficacy has been compromised by the emergence of various *mcr* genes (6), most of which are located on plasmids in *Enterobacteriaceae* isolated from both animals and humans (7, 8).

Nontyphoidal Salmonella is one of four major causative agents of diarrheal diseases globally, with most cases of salmonellosis being mild. However, life-threatening infections may develop (9). Serotype is strongly associated with disease severity as well as prevalence, and 2,650 serovars of Salmonella enterica have been described (10). S. enterica serovars Typhimurium and Enteritidis have been identified as the most common culprits of invasive human Salmonella infection. Furthermore, strains of *S*. Typhimurium and its monophasic variant 1,4,[5],12:i:–, especially those of sequence type 34 (ST34), play an important role in the transmission of *mcr* genes from food animals to human populations, even if colistin is not used to treat salmonellosis (11–14).

While the mode of the spread of *mcr* genes in *Enterobacteriaceae*, especially Escherichia coli, has been described previously (7), there has been no large and systematic study to elucidate *mcr* gene transmission in Salmonella (7, 15). *mcr* genes are transferred mainly horizontally via two distinct mechanisms: (i) plasmid dissemination, which is reflected by the same plasmid type and different STs (16, 17), and (ii) the dissemination of insertion sequences (ISs) or transposons between different plasmid types (18, 19). The nested nature of these mobile genomic elements resembles the Russian doll model (20).

Here, we used a large and well-characterized collection of 140 *mcr*-carrying Salmonella isolates generated from the China National Foodborne Disease Surveillance and Outbreak program (our unpublished data) to examine the modes of the spread of *mcr* genes in the clinical setting over a 10-year period. We explored the modes of the spread of *mcr* genes at a high resolution by relating the whole-genome sequences to plasmid traits (short reads aligned to a reference plasmid). We sought to demonstrate a superior method for elucidating the modes of *mcr* gene dissemination occurring in nontyphoidal Salmonella isolates from diarrheal outpatients in China, in a bid to improve the control of transmission between animals and humans.

## RESULTS

### Salmonella isolates collected from patients from 29 provinces across China between 2011 and 2020.

**FIG 1:**
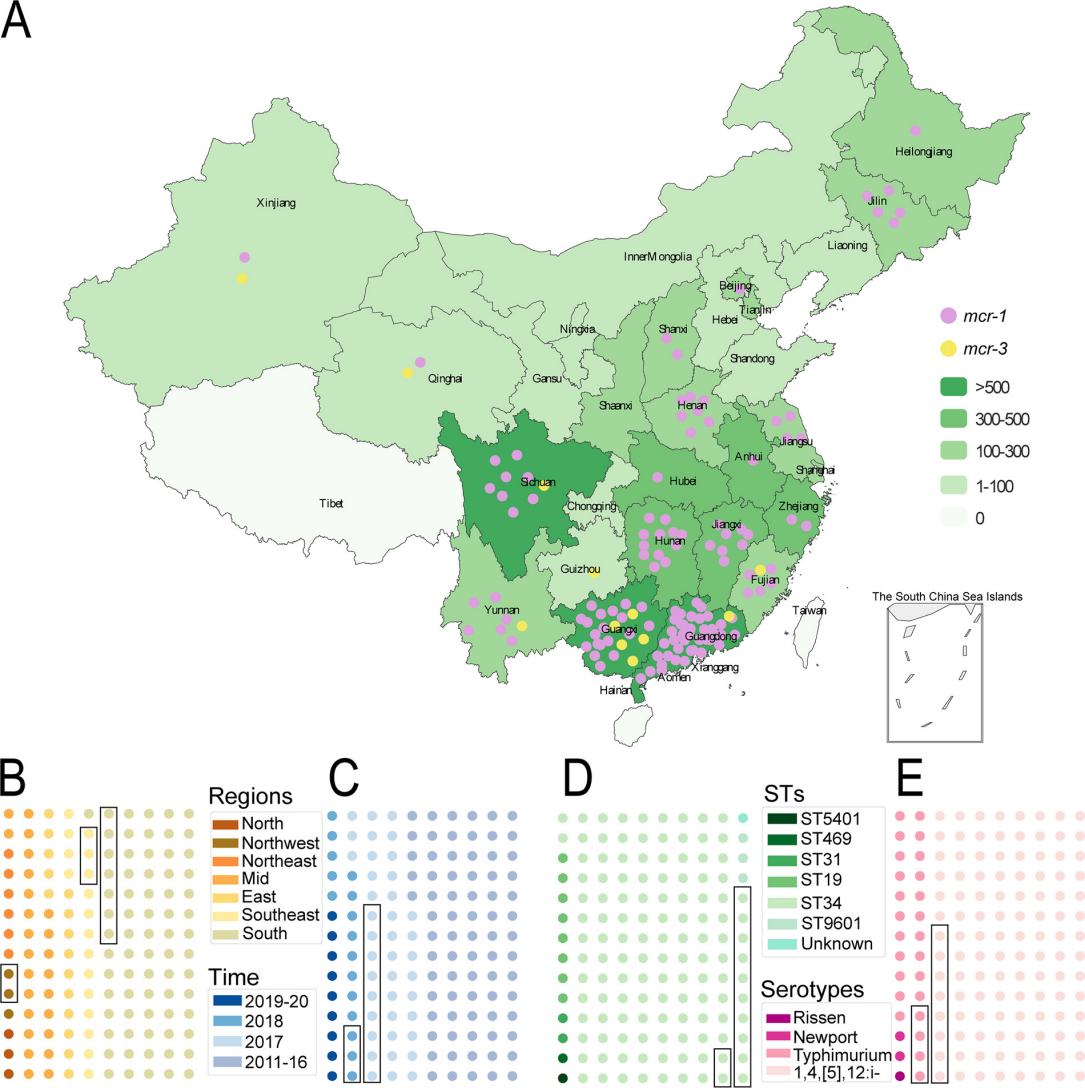
Distribution of Salmonella isolates included in this study across China, 2011 to 2020. (A) Green heat map showing the numbers of Salmonella isolates from different provinces (*n* = 7,106). Plum dots in the map indicate *mcr-1*-positive Salmonella isolates, and yellow dots indicate *mcr-3*-positive isolates, with one dot per isolate. (B to E) Regions, times, STs, and serotype patterns of 140 *mcr*-positive isolates. The dots in the box indicate *mcr-3-*positive isolates, whereas the other dots refer to *mcr-1*-positive isolates.

All isolates were obtained from the National Molecular Tracing Network for Foodborne Disease Surveillance (TraNet) in China. From 2011 to 2020, a total of 7,106 Salmonella isolates were obtained from 29 provinces (Fig. 1A). The majority of the isolates were from Guangdong (17.3% [1,228/7,106]), Sichuan (10.2% [723/7,106]), and Guangxi (7.8% [555/7,106]) (Fig. 1A). Antimicrobial resistance genes (ARGs) in Salmonella isolates were identified using ResFinder (version 4.0) (21), with the identity and coverage cutoff values set to 90% and 60%, respectively. In total, we identified 128 *mcr-1*-positive and 12 *mcr-3*-positive isolates, distributed across 19 provinces. The *mcr-1*-positive isolates were obtained mainly from Guangdong (31.3% [40/128], 2017 to 2018), Guangxi (14.8% [19/128], 2016 to 2019), and Hunan (13.3% [17/128], 2015 to 2019). *mcr-3*-positive isolates were obtained mainly from Guangxi (41.7% [5/12], 2017 to 2018) (Fig. 1B and C). All 7,106 Salmonella isolates could be divided into 151 STs based on seven host genes and 107 distinct serotypes based on the presence of O and H antigen loci.

Further analysis indicated that the *mcr-1* and *mcr-3* genes were host specific and present in limited STs and a small number of serotypes. *mcr-1*-positive isolates (*n* = 128) belonged to 7 different STs, including ST34 (87.5% [112/128]), ST19 (6.25% [8/128]), ST31 (1.6% [2/128]), ST469 (0.8% [1/128]), ST5401 (0.8% [1/128]), and an unknown ST (0.8% [1/128]). All *mcr-3*-positive isolates (*n* = 12) belonged to ST34 (Fig. 1D and E). ST34 isolates were the predominant hosts of *mcr-1* and *mcr-3* genes (88.6% [124/140]), which were widespread in most of the collection regions between 2011 and 2020. Uncommon STs were detected mostly in South China from 2016 to 2018 (Fig. 1D; see also Fig. S1A and C in the supplemental material). *mcr-1* and *mcr-3* genes were present in isolates of only 4 of 107 serotypes. The most prevalent among these was 1,4,[5],12:i:– (*mcr-1*, 81.3% [104/128], 2014 to 2019; *mcr-3*, 66.7% [8/12], 2017 to 2018), followed by *S*. Typhimurium (*mcr-1*, 16.4% [21/128], 2015 to 2020; *mcr-3*, 33.3% [4/12], 2017 to 2018). Similar to the STs, the above-mentioned two serotypes were observed across the majority of the collection areas. In addition, two *S*. Newport isolates and one *S*. Rissen isolate harbored the *mcr-1* gene, obtained from South China in 2016 and 2017, respectively (Fig. 1E and Fig. S1B and D). Detailed information regarding the province distributions of 7,106 Salmonella isolates and 140 *mcr*-positive Salmonella isolates is summarized in Table S7, and the metadata for the 140 *mcr*-positive Salmonella isolates is summarized in Table S8.

### High diversity of genetic environments containing *mcr* genes.

**FIG 2:**
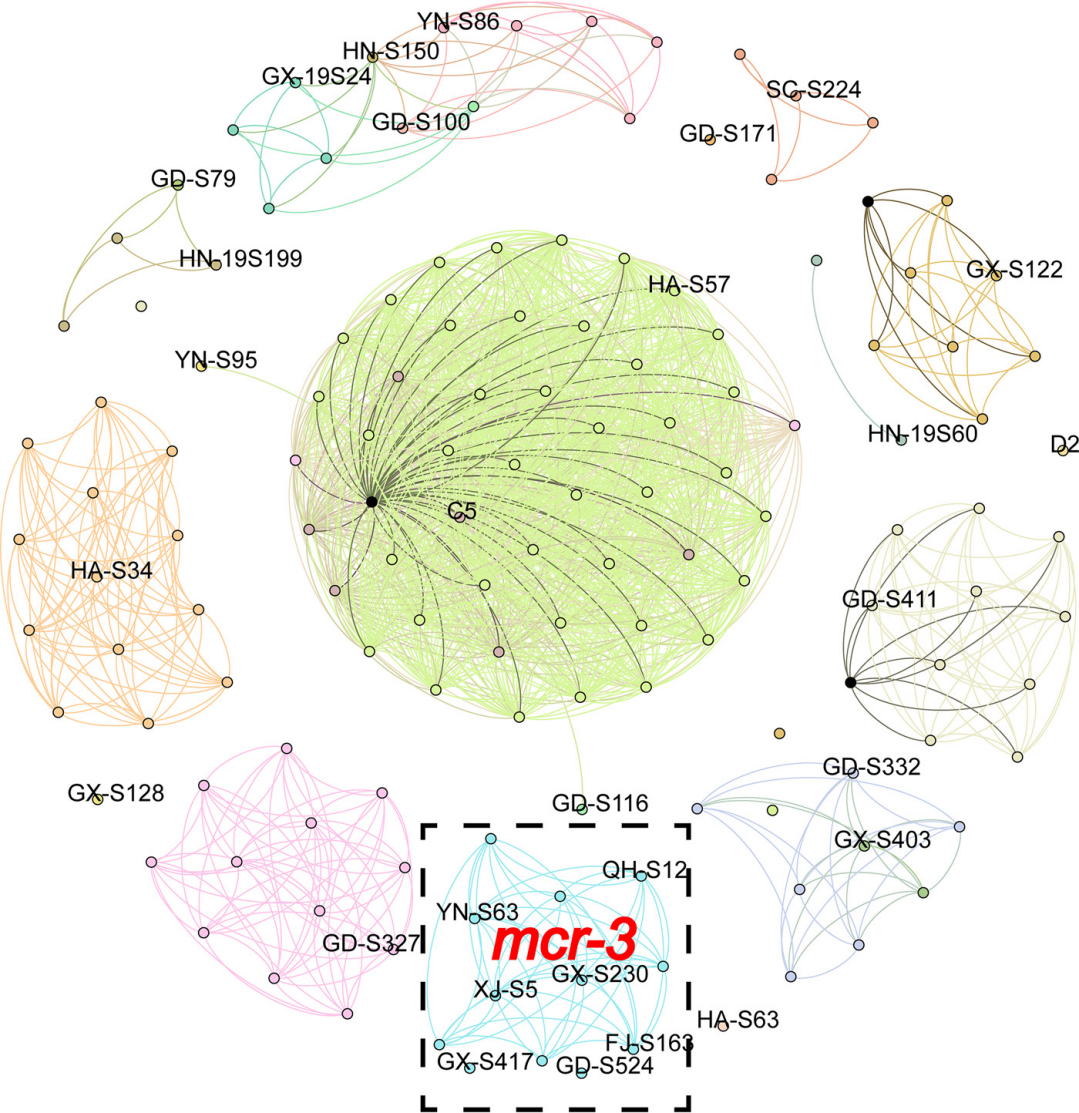
*mcr*-carrying flanking sequence community network. The *mcr*-flanking sequence network uses a threshold of 0.01. Each group (23 groups in total) has a unique color based on the ANI. Isolates for long-read sequencing are labeled by the isolate name. The isolates inside the dotted box refer to *mcr-3*-flanking sequences, and those outside the dotted box refer to *mcr-1*-flanking sequences.

To characterize the genetic environments of *mcr* genes, all *mcr*-carrying contigs were extracted from the *de novo*-assembled draft genomes. The lengths of the *mcr*-carrying contigs generated by Illumina sequencing ranged from 2,191 to 206,978 bp. Based on the average nucleotide identity (ANI), contigs were clustered into groups using three methods (see Materials and Methods) (Table S1) (22). As a result, *mcr*-carrying contigs were divided into 23 different groups, with 22 groups harboring *mcr-1* genes and only 1 group harboring *mcr-3* genes. To comprehensively analyze the various genetic environments of *mcr-1* and *mcr-3* genes and obtain the complete chromosomes or plasmids containing these genes for further analysis, we selected one representative isolate from each *mcr-1*-carrying group for long-read sequencing. Due to the short reads and the high number of insertion sequences (Fig. S2), the *mcr-3-*carrying contigs were too short to be grouped with high resolution. Thus, we selected seven cross-sectional isolates (GX-S230, GD-S534, YN-S63, FJ-S163, GX-S417, QX-S12, and XJ-S5) based on different contig lengths for long-read sequencing. All isolates selected for long-read sequencing are labeled in Fig. 2.

To test the rationale for the selection of representative isolates, *mcr*-flanking sequences, which included 3,000 bp upstream of the *mcr* gene, the *mcr* gene, and 2,000 bp downstream, rather than *mcr* contigs, were subjected to analysis owing to the uneven length of the latter. The distance of *mcr-*flanking sequences was estimated using Mash (version 2.3) (23) based on *k-*mers (see Materials and Methods) (Table S2). We used a similarity of 0.01 (~0 to ~1, with values of 0 being identical sequences and 1 being dissimilar sequences) as a threshold in the *mcr*-flanking sequence network. This involved removing all edges above a fixed Mash threshold. Network visualization at this threshold with different groups in unique colors is shown in Fig. 2. Altogether, we obtained 11 groups (more than two isolates), including 10 for *mcr-1*-flanking sequences and 1 for *mcr-3*-flanking sequences. All 11 groups contained at least one of the representative isolates selected as described above. In contrast, three out of five *mcr-1*-flanking groups contained only one selected isolate (Fig. 2). Thus, it is plausible to assume that the genetic environments containing *mcr* genes of the selected isolates could be representative of Salmonella isolates in our study.

### *mcr* genes existed mainly on a variety of plasmids with different characteristics.

To better understand the locations of *mcr-1* and *mcr-3* genes for the selected isolates, we assembled the long-read sequencing data with the short reads using Unicycler (version 4.18) (24) for 29 representative isolates (Table S3). In total, we obtained 22 complete *mcr-1*-carrying plasmids (defined as <350,000 bp with a circular topology [25]), ranging from 33,309 to 292,357 bp. In addition, *mcr-3* genes were present on five plasmids, ranging from 88,129 to 168,147 bp, as well as two chromosomes with lengths of 4.88 and 4.90 Mb. For unselected isolates, we aligned the short sequence reads from all 140 isolates to each of the plasmid sequence assemblies since the majority of the *mcr-1* or *mcr-3* genes were located on plasmids. Generally speaking, when mapping short reads to complete *mcr*-positive plasmids, the align value was highest, close to 100%. For other isolates, if the align value was >95%, we presumed that these were highly similar plasmids. If the align value was >80% and <95%, we presumed that they had similar plasmid backbones (Table S9). Importantly, this approach cannot rule out insertions or rearrangements related to the reference plasmid (22). Nevertheless, it provided approximate information for the plasmids present in Salmonella isolates without long-read sequencing data.

**FIG 3:**
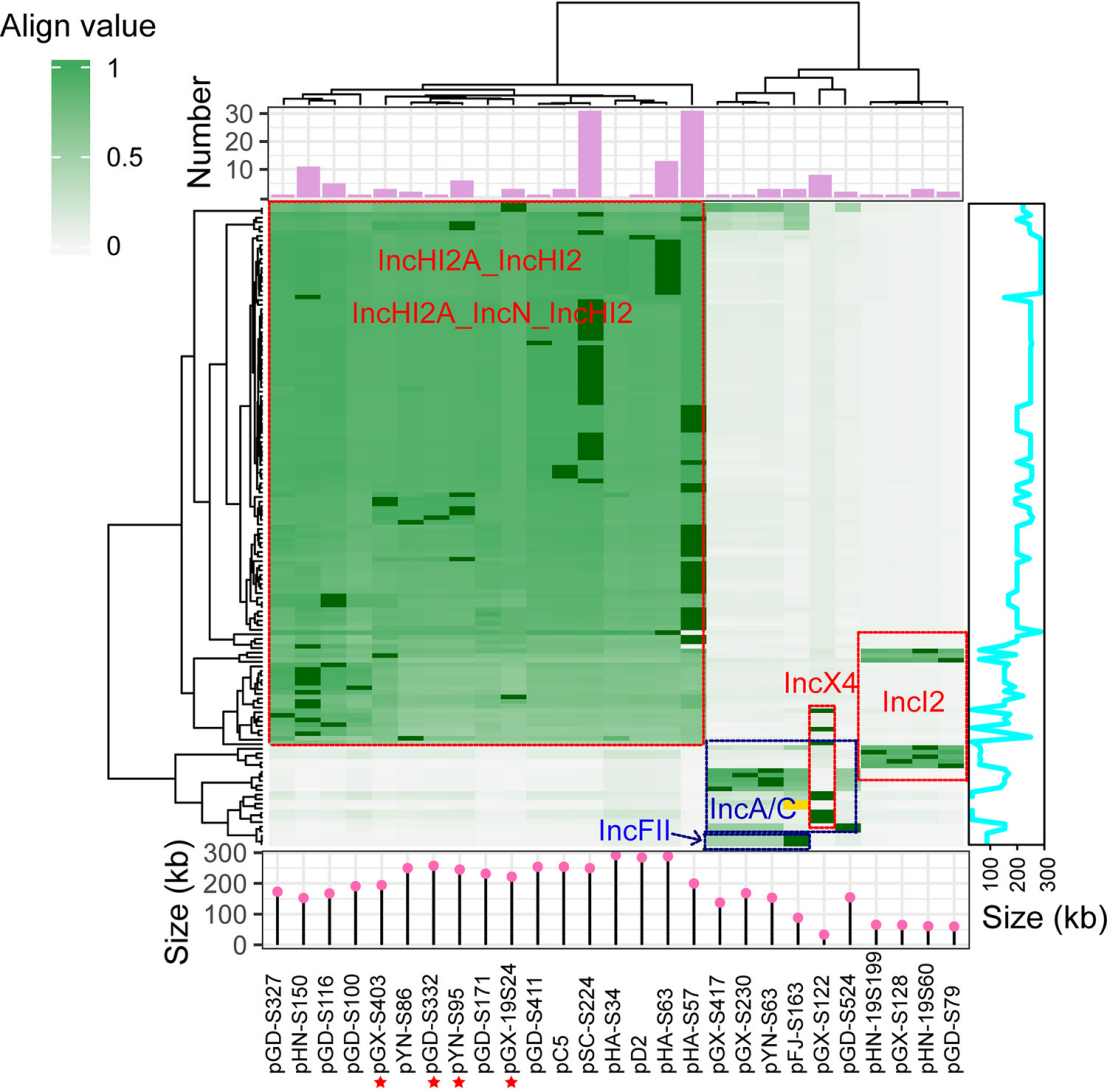
High prevalence of *mcr*-positive plasmids across *mcr*-positive Salmonella isolates. The heat map shows the align values of short reads from 140 *mcr*-positive isolates aligned to 27 reference plasmids from hybrid assemblies (the row is the reference plasmid; the column is the *mcr*-positive isolate). Dark green refers to predicted plasmids with the highest align values. Gold refers to the *mcr-3* gene in the chromosome. The plum bar plot shows the prevalence of reference plasmids. The hot-pink bar-dot plot shows the lengths of 27 reference plasmids from hybrid assemblies. The cyan line plot shows the lengths of the predicted plasmids in 140 *mcr*-positive isolates. Red stars refer to IncHI2A_IncN_IncHI2 plasmids.

Consequently, we found that pHA-S57 and pSC-S224 (from isolates HA-S57 and SC-S224) were the most predominant *mcr-1*-carrying plasmids, and both of them were present in 31 Salmonella isolates (21.22% [31/128]). In addition, pHA-S63 (from HA-S63) and pHN-S150 (from HN-S150) were present in 13 isolates (10.16% [13/128]) and 11 isolates (8.59% [11/128]), respectively. pYN-S63 and pFJ-S163 (from YN-S63 and FJ-S163) were the main *mcr-3*-carrying plasmids, with both being present in 3 isolates (25.00% [3/12]). The genetic environments of *mcr-3* carried on chromosomes in two isolates (QH-S12 and XJ-S5) showed high identity to pFJ-S163, indicating the possibility that chromosomal *mcr-3* was from a pFJ-S163-like plasmid (Fig. 3 and Fig. S1). These results suggested that *mcr-1* and *mcr-3* genes were transferred mainly by various diverse plasmids.

**FIG 4:**
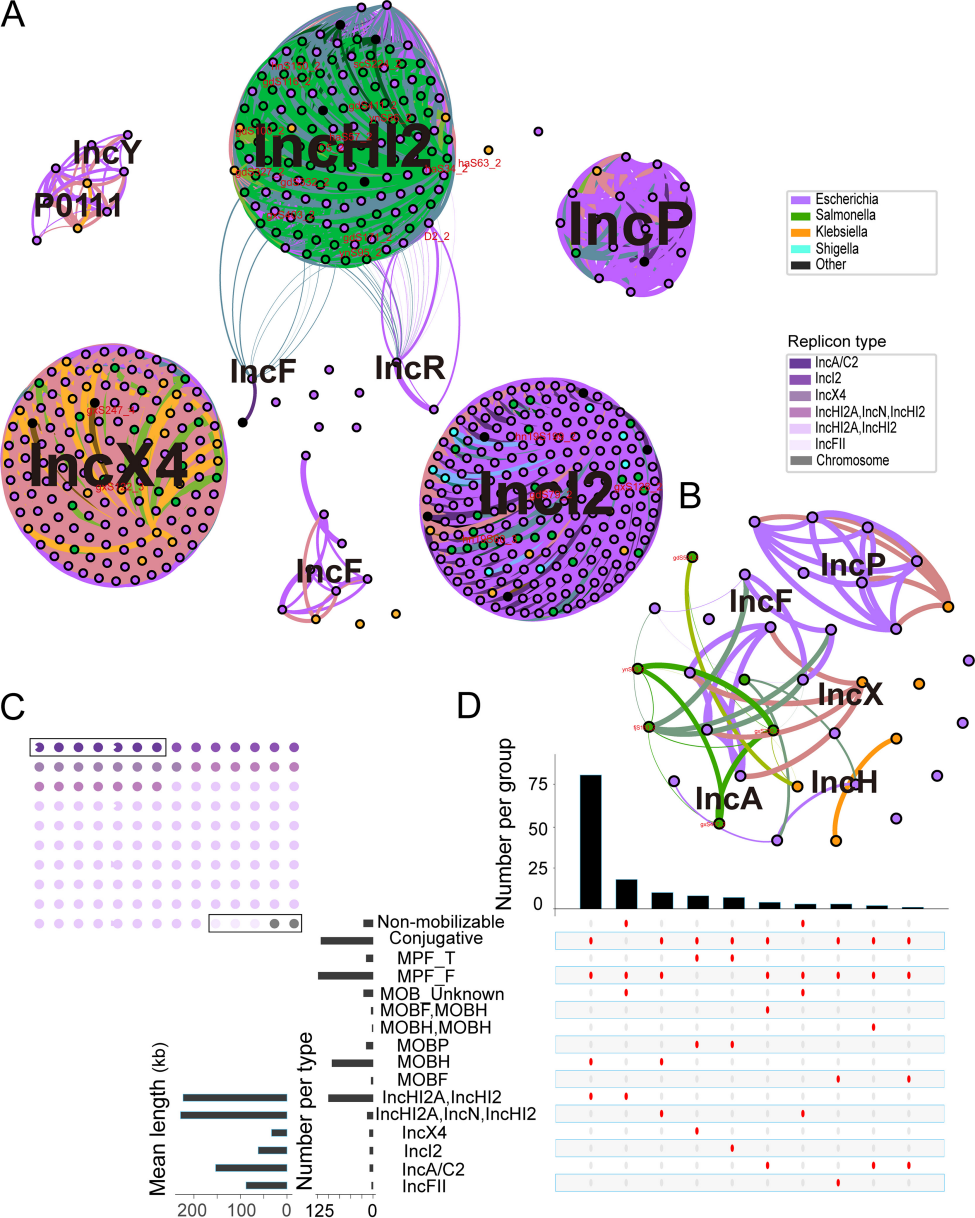
Diversity of plasmids carrying *mcr* genes from our study and the GenBank database. (A) Diversity of plasmids carrying the *mcr-1* gene (*n* = 518). The network sets a value of 0.01 as the similarity threshold. The bacterial hosts with various plasmids based on the replicon types are displayed with unique colors. The plasmids sequenced in our study are labeled in yellow. (B) Diversity of plasmids carrying the *mcr-3* gene (*n* = 36). The network sets a value of 0.01 as the similarity threshold. The bacterial hosts with various plasmids based on the replicon types are displayed with unique colors. The plasmids sequenced in our study are labeled in red. (C) Waffle plot of the replicon types of the 140 *mcr*-positive isolates according to predicted plasmids. The dots in the box refer to *mcr-3*-positive isolates, whereas the other dots refer to *mcr-1*-positive isolates. (D) Intersection plot of the combination of replicon types, MOB, MPF, and mobility found in the set of predicted plasmids (*n* = 140).

Furthermore, to determine additional features of the above-mentioned plasmids, we characterized them based on replicon types, the relaxases for mobilization (MOB), MPF (mating pair formation) complexes, and mobility. All of the plasmids belonged to six incompatibility groups. IncHI2A_IncHI2 (100/128 [78.13%]; mean plasmid length, 229.78 kb), IncHI2A_IncN_IncHI2 (13/128 [10.16%]; mean plasmid length, 229.51 kb), IncX4 (8/128 [6.25%]; mean plasmid length, 33.31 kb), and IncI2 (7/128 [5.47%]; mean plasmid length, 62.66 kb) were the major *mcr-1*-positive plasmids (Fig. 3 and Fig. 4C and D), whereas IncA/C (7/12 [58.33%]; mean plasmid length, 153.30 kb) and IncFII (7/128 [5.47%]; mean plasmid length, 88.13 kb) were the major *mcr-3*-positive plasmids (Fig. 3 and Fig. 4C and D). Plasmids MOBH (92/140 [65.71%]) and MPFF (123/140 [87.86%]) were the most prominent. A considerable proportion of the plasmids were predicted to be conjugative (118/140 [84.29%]), consistent with the presence of one or more MOB genes, with the combination of IncHI2A_IncHI2_MPFF_MOBH (82/140 [58.57%]) being the most common, while a smaller number were predicted to be nonmobilizable (22/140 [15.71%]), without MOB-related genes (Fig. 4D).

In order to explore a wider range of differences upstream and downstream of the *mcr-1* and *mcr-3* genes, we extracted the regions 10,000 bp upstream and downstream of the *mcr-1* and *mcr-3* genes from 29 representative isolates in order to analyze differences in the genetic environment between different plasmids. There were 19 different types: types 1 to 3 and types 7 to 9 for IncHI2A_IncHI2, types 4 to 6 for IncHI2A_IncN_IncHI2, type 10 for IncX4, types 11 to 13 for IncI2, types 14 to 17 for IncA/C, type 18 for IncFII, and type 19 for the *mcr*-positive chromosome. The genetic environment of *mcr-1* was less conserved than that of *mcr-3*. A total of 90.91% (20/22) of the isolates contained *mcr-1-*hp (hypothetical protein), and one-half of the isolates (11/22 [50%]) contained IS*Apl1–mcr-1-*hp, while all *mcr-3*-positive isolates (*n* = 7) contained *mcr-3–dgkA–*IS*Kpn40*, 6 out of 7 contained Tn*As2–mcr-3–dgkA–*IS*Kpn40*, and 3 out of 7 contained IS*26–*Tn*As2–mcr-3–dgkA–*IS*Kpn40*. Furthermore, 36.36% (8/22) of the *mcr-1* genes coexisted with one copy of an IS, and 22.73% (5/22) did not coexist with an IS, while 100.00% (7/7) of the *mcr-3* genes coexisted with at least three copies of an IS and one copy of a transposon. We found that the genetic environment of nonmobilizable plasmids showed a striking similarity to that of mobilizable plasmids and possessed one copy of IS*Apl1*, except for pGX-S403 (Fig. S1). Furthermore, nonmobilizable plasmids were usually shorter than conjugative ones, ranging from 152,570 to 194,331 bp (mean plasmid length, 164,857 bp) (Fig. 4D). Taken together, the above-described data indicate that the *mcr*-positive plasmids in Salmonella are highly diverse.

### The reduction of *mcr*-positive isolates was related to their decreased prevalence in IncHI2A_IncHI2 plasmids.

**FIG 5:**
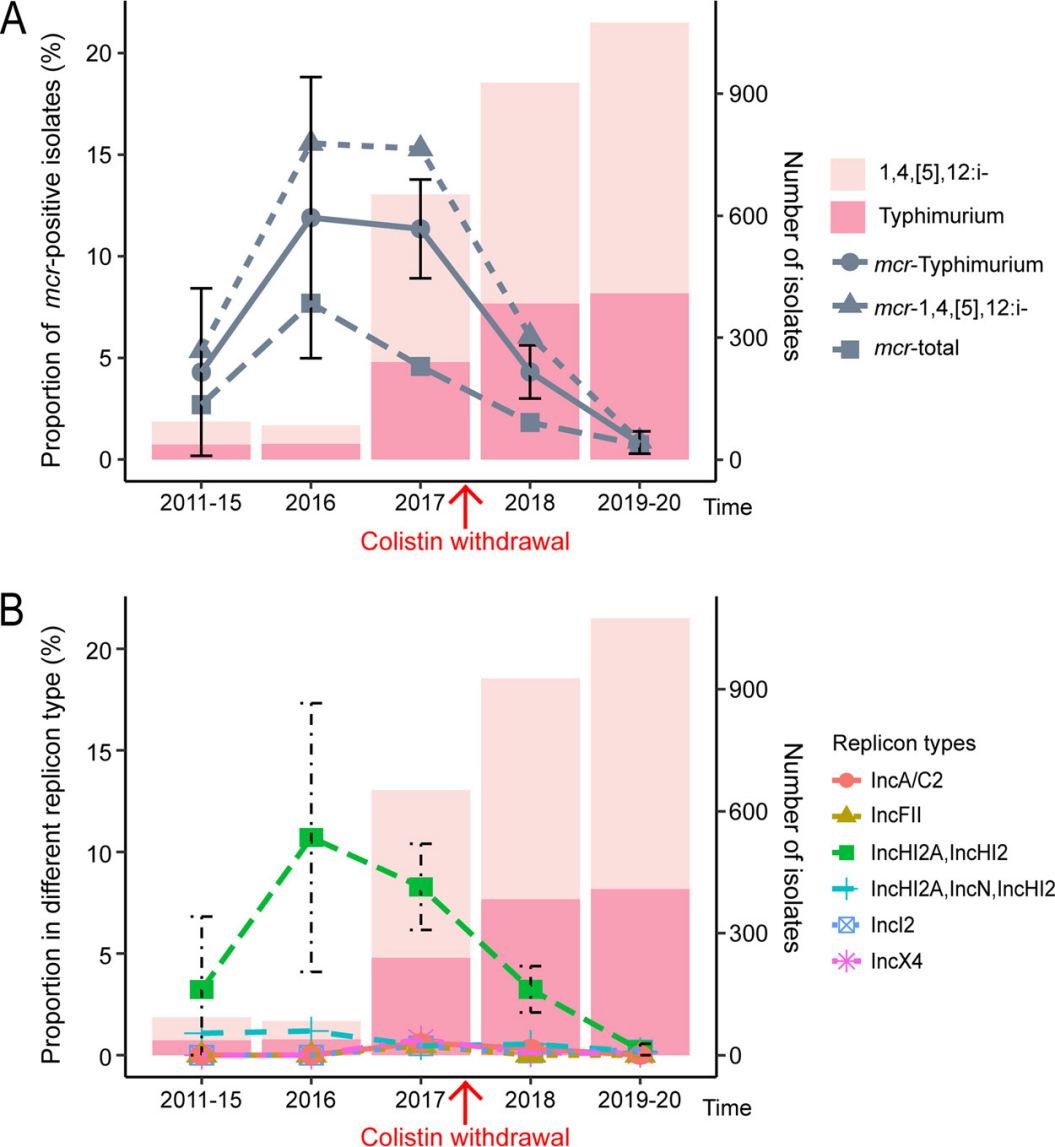
Proportions of *mcr*-positive isolates and their replicon types for 1,4,[5],12:i:– and *S.* Typhimurium at different time points. (A) Three line plots showing the proportion of 1,4,[5],12:i:–, *S.* Typhimurium, or total *mcr*-positive isolates. The stacked bar plot shows the numbers of *mcr*-positive isolates of 1,4,[5],12:i:– and *S.* Typhimurium. (B) Replicon types of *mcr-1*- or *mcr-3*-carrying plasmids in 1,4,[5],12:i:– and *S*. Typhimurium isolates.

A significant reduction in *mcr*-positive Salmonella isolates has been observed since 2017 (Fig. 5A). Previous studies suggested that the ban on the use of colistin as a growth promoter resulted in a significant decrease in the proportion of *mcr*-positive E. coli isolates from both animals and humans (26, 27). Our study indicated that the overall proportion of *mcr* genes in 1,4,[5],12:i:– and *S*. Typhimurium isolates significantly decreased from 11.35% (74/652) in 2017 to 4.31% (40/927) in 2018 (*P* < 0.0001) and 0.79% (8/1,017) in 2019 (*P* < 0.0001) (Fig. 5A). These findings indicated that the implementation of the colistin ban policy not only reduced the detection of *mcr* genes in E. coli isolates from animals and humans but also had a similar effect on *mcr* genes in Salmonella isolates from humans (Table S10).

To characterize the molecular basis of this reduction in *mcr-*positive isolates, we further characterized the replicon types of *mcr-1*- and *mcr-3*-positive Salmonella isolates at different times. We found that only IncHI2A_IncHI2 plasmids changed over time: 3.23% (3/93) from 2011 to 2015, 10.71% (9/84) in 2016, 8.28% (54/652) in 2017, 3.24% (30/927) in 2018, and 0.28% (3/1,075) from 2019 to 2020. No other plasmids changed significantly. Moreover, the changing trend in plasmid replicon types over time was highly consistent with the changing trend in *mcr* detection in dominant serotypes (Fig. 5B). Therefore, we may be able to conclude that the spread of IncHI2A_IncHI2 plasmids was the main cause of the variation in *mcr* detection (Table S11).

### The single-population structure of Salmonella carrying *mcr* in China.

**FIG 6:**
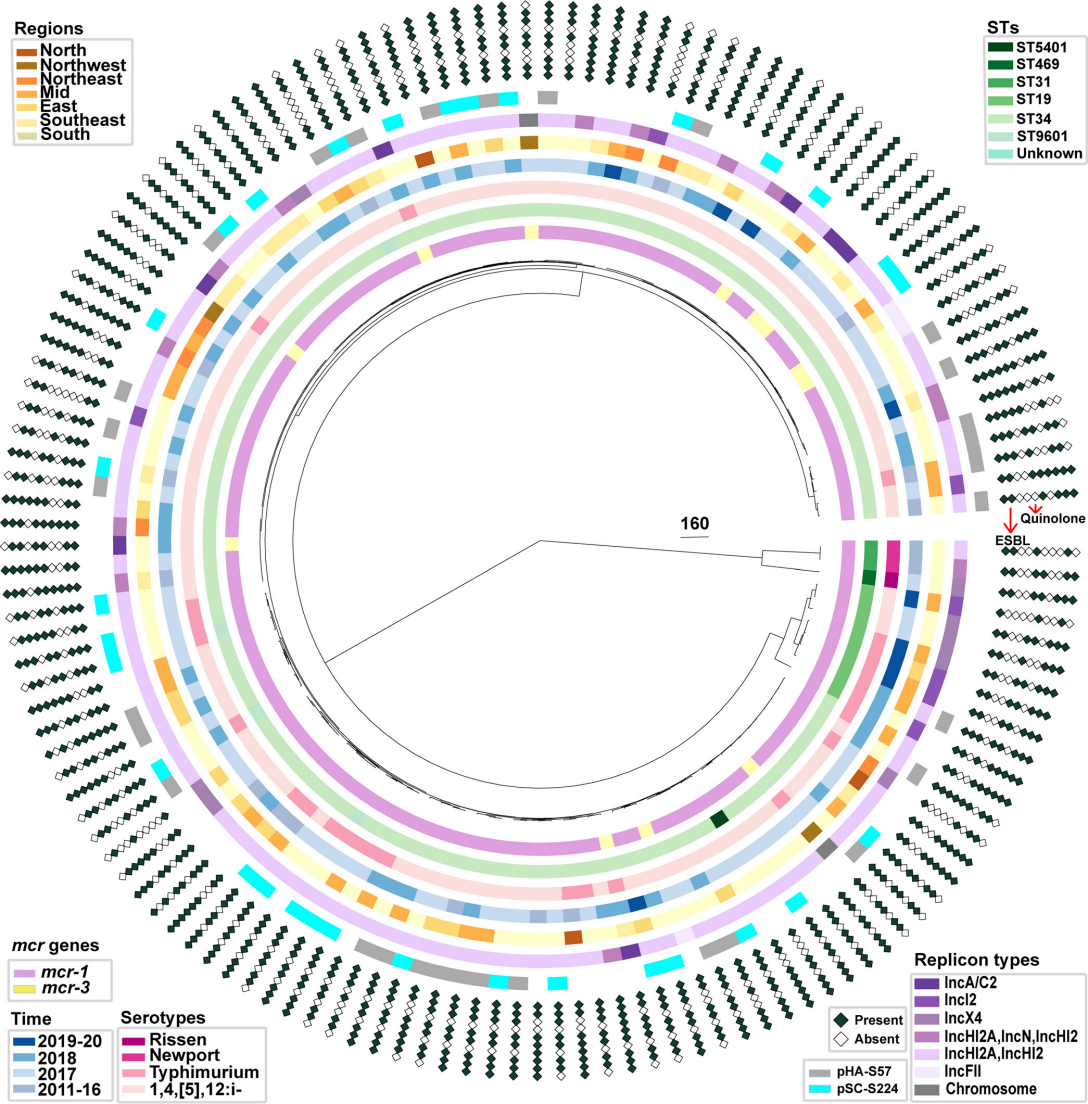
Phylogenetic analysis of *mcr*-carrying Salmonella isolates. Shown are the genetic relationships among Salmonella isolates (*n* = 140). Phylogenetic tree construction is based on 330 cgMLST loci. The circular columns toward the outer part of the tree indicate the *mcr* genes, STs, serotypes, times, regions, replicon types, and prevalences of plasmids pHA-S57 and pSC-S57. The outermost 10 circles indicate the patterns of resistance to antimicrobial classes. The inner to outer diamonds indicate the presence or absence of aminoglycoside, ESBL, fosfomycin, macrolide, phenicol, quinolone, rifampicin, sulfonamide, tetracycline, and trimethoprim ARGs.

To explore the evolutionary relationships of the same popular plasmids, we selected the most prevalent predicted plasmids in our data set, namely, pHA-S57 and pSC-S224. In addition, we combined the data from the core-genome multilocus sequence typing (cgMLST) phylogenetic tree, STs, serotypes, times, regions, replicon types, and ARG profiles. cgMLST loci were predicted via SISTR_cmd, and differences between isolates were calculated to construct the cgMLST phylogenetic tree based on 330 loci (see Materials and Methods) (28). The ARG phenotypes for 140 assemblies were predicted using ABRicate (version 1.0.1) in the ResFinder database (29) (see Materials and Methods). The inferred cgMLST tree had two main branches: one large branch comprised of 1,4,[5],12:i:– and *S*. Typhimurium isolates of mainly ST34 and another large branch comprised of *S*. Newport and *S*. Rissen isolates. In the large clades, only a few cgMLST loci differed, and the prevalent *mcr*-positive plasmids were scattered in the large branch. This observation suggests that the same plasmid was mobilized between similar cgMLST settings but different chromosomal settings (Fig. 6).

Furthermore, to investigate the coexistence of *mcr* genes with other ARGs, we compared the ARG profiles of 140 assembles and found that *mcr* genes coexisted with the two major types of ARGs threatening human health: extended-spectrum β-lactamase (ESBL) genes (*bla*_CTX-M-14_, *bla*_CTX-M-55_, *bla*_CTX-M-64_, *bla*_CTX-M-65_, *bla*_OXA-10_, *bla*_OXA-1_, *bla*_TEM-104_, *bla*_TEM-135_, and *bla*_TEM-1B_) and quinolone resistance genes [*aac(6′)-Ib-cr*, *oqxA*, *oqxB*, *qnrS1*, and *qnrS2*]. Other ARGs were also noted, including aminoglycoside [*aac(3)-IId*, *aac(3)-IVa*, *aac(6′)-Iaa*, *aadA16*, *aadA1*, *aadA2*, *aadA2*, *ant(3″)-Ia*, *aph(3″)-Ib*, *aph(3′)-Ia*, *aph(4)-Ia*, and *aph(6)-Id*], fosfomycin (*fosA3*), macrolide [*mph*(A), *lnu*(F), and *mef*(B)], phenicol (*catA2*, *catB3*, *cmlA1*, and *floR*), rifampicin (*ARR-3*), sulfonamide (*sul1*, *sul2*, and *sul3*), tetracycline [*tet*(A), *tet*(B), and *tet*(M)], and trimethoprim (*dfrA12*, *dfrA14*, *dfrA27*, and *dfrA5*) resistance genes (Fig. 6). In order to explore the colocalization status of *mcr* genes and other ARGs, we compared the ARG profiles of 27 completely sequenced plasmids (Fig. S2). We found that 77.3% (17/22) of the *mcr-1*-positive plasmids contained other ARGs, as did 100% (5/5) of the *mcr-3*-positive plasmids. Furthermore, 9.1% (2/22) of the *mcr-1*-positive plasmids carried ESBL and quinolone ARGs, while 40% (2/5) of the *mcr-3*-positive plasmids carried ESBL and quinolone ARGs (Fig. S2). In addition, the antimicrobial resistance of *mcr*-positive Salmonella isolates to clinical antibiotics was tested. More than 90% of the isolates were resistant to tetracycline, and the rates of resistance to lactam antibiotics, such as ampicillin and ampicillin-sulbactam, were 80% and 95%. A total of 57% of the isolates were resistant to colistin, and 23% of the isolates were resistant and 46% were intermediate to ciprofloxacin. A total of 8% of the isolates were resistant to azithromycin (Fig. S5). Taken together, the above-described findings suggest that a limited number of high-risk clones (ST34) are responsible for most of the *mcr* gene transfer events, and multiple ARGs often coexist or colocalize with *mcr*-positive plasmids, which allows the relatively easy spread of MDR pathogens from animals to humans.

## DISCUSSION

Data on the prevalence of *mcr* genes in food animals, wild animals, pets, humans, and the environment indicate that *Enterobacteriaceae* are the most likely primary hosts (8), with *mcr*-carrying plasmids deposited in the GenBank database (see Fig. S3 in the supplemental material) indicating similar results. That is, most *mcr*-positive plasmids are present in E. coli, followed by Klebsiella pneumoniae or Salmonella. It is therefore reasonable to assume that *Enterobacteriaceae* fulfill important roles during *mcr* gene circulation. However, there are still gaps in the understanding of the mode of *mcr* transmission of Salmonella from patients (30). In this study, we investigated the nationwide spread of the *mcr-1* and *mcr-3* genes in nontyphoidal Salmonella isolates over 10 years.

*mcr* genes are located mainly on plasmids (16). To obtain complete plasmid sequences and clarify the mechanism of the transmission of *mcr* genes, previous studies usually combined long-read and short-read sequencing (30). Considering the cost when too many isolates are to be sequenced, we cannot subject all isolates to long-read sequencing. Thus, representative or interesting isolates are generally selected for long-read sequencing depending on the sampling time and location or length of contigs. However, this approach is stochastic and not suitable for all scenarios. Here, we first extracted *mcr* contigs and *mcr*-flanking sequences at the same time, considering that the lengths of different contigs vary greatly. We then calculated the ANI for groupings based on the complete *mcr* contig or *mcr*-flanking sequences with the threshold set at 80% or 95%. Representative isolates were then selected for long-read sequencing so that the assembly of the circular *mcr* plasmid data set contained all *mcr*-positive plasmids of Salmonella. Finally, taking the assembly circular *mcr* plasmid assembly data set as a reference, we obtained *de novo mcr* plasmid predictions for the set of 140 *mcr-1* or *mcr-3*-positive samples with associated short reads. Compared with other plasmid prediction methods, this method can more accurately predict the plasmid backbone rather than just defining the plasmid type (31).

We tried to explore the similarities and differences between the plasmids obtained by sequencing and those in the GenBank database. We downloaded 803 plasmids carrying *mcr-1* and 59 carrying *mcr-3* from the GenBank database before 19 January 2022. Appending our sequenced plasmids with *mcr* genes, we obtained 518 plasmids carrying *mcr-1* and 36 carrying *mcr-3*. Distances between pairs of plasmids were calculated using Mash triangle (23) (see Materials and Methods), and its output was used to create an unoriented network (Table S6). To achieve appropriate sparsification in plasmid networks and analyze the relationship between our sequenced plasmids and plasmids in GenBank, we used a value of 0.01 as the similarity threshold in our plasmid network. Network visualization of the host bacteria is shown in Fig. 4A and B, and the sequenced plasmids are labeled with names defined by us. Our analysis revealed that all sequenced plasmids were clustered with previously reported plasmids (Fig. 4A and B) (32). For *mcr-1*-positive plasmids, we show that *mcr-1* was acquired by plasmids of various replicon types, such as the IncHI2A_IncHI2 plasmid type that was found for every ST in our study (Fig. 6), while IncI2 was the most common *mcr-1*-carrying plasmid type in the GenBank database, and IncHI2 was the third most common (Fig. S4A). In our study, IncI2 (7/128 [5.47%]) was less common. In 2019, X. Lu et al. reported a retrospective survey of *mcr-1*-harboring Salmonella isolates from diarrheal outpatients in Shanghai, China, from 2006 to 2016, with IncI2, IncHI2, and IncX4 being identified as the main replicon types (30). For *mcr-3*-positive plasmids, IncFII (Fig. S4B) was the most prevalent replicon type in both our study and the GenBank database, as previously reported (14, 15).

Previous studies have shown that Salmonella is transmitted predominantly via commercially produced food contaminated by animal feces (9). Furthermore, *mcr* genes in patients are most likely derived from food animals such as pigs (30). In addition, colistin is used in humans only when no other effective drug is available, let alone for the treatment of diarrheal diseases caused by nontyphoidal Salmonella (13). Therefore, we hypothesize that the colistin ban and its decreased use in food animals may lead to changes in the proportion of *mcr*-positive Salmonella isolates detected from diarrheal patients. Indeed, our results suggest that the *mcr* abundance is changing in isolates of Salmonella Typhimurium and its variant 1,4,[5],12:i:– from diarrheal patients in China. This finding is consistent with those of Wang et al., who observed that the cessation of colistin use as a feed additive had a significant effect on reducing colistin resistance in both animals and humans in China (26). It is also in agreement with the findings of Shen et al., who described rapid, ecosystem-wide declines in the *mcr-1* prevalence and *mcr-1*-positive E. coli isolates after the use of colistin in animal feed was banned in China (27). Furthermore, our results also suggest that the reduced prevalence of *mcr*-positive Salmonella isolates is associated mainly with the IncHI2A_IncHI2 plasmid type (Fig. 5B).

We acknowledge several limitations of our study. First, some contigs obtained from whole-genome sequencing (WGS) assemblies were <6,000 bp, and we could not cluster them into groups with high accuracy. Besides, we leave annotation information and other factors out of consideration, focusing on the ANI alone, and thus, some groups may be redundant. Second, our *mcr*-positive Salmonella strains were isolated mainly in 2017 and 2018, resulting in an uneven time distribution. Third, we did not analyze short-read sequencing data for *mcr*-positive Salmonella isolates in public databases and combined only complete plasmid sequences in the GenBank database, possibly ignoring the relationship between the plasmid and the host or source.

In conclusion, our study revealed that the *mcr-1* and *mcr-3* genes were transmitted in similar genome settings (high-risk clones of ST34) but through plasmids of various replicon types. In addition, we found that the policy of banning colistin use in food animals can lead to a decrease in the prevalence of *mcr*-positive Salmonella isolates from diarrheal patients, related mainly to IncHI2A_IncHI2 plasmids. Furthermore, we provide a new approach for discerning the dynamics and the mode of dissemination of *mcr* genes, which can be applied to study other AMR genes such as *bla*_NDM_ in *Enterobacteriaceae*.

## MATERIALS AND METHODS

### Dividing *mcr*-positive contigs into different groups.

All contigs carrying *mcr* genes were extracted. *mcr*-flanking sequences, which include 3,000 bp upstream of the *mcr* gene, the *mcr* gene, and 2,000 bp downstream of the *mcr* gene, were extracted using SAMtools (version 1.14) (33) and bedtools (version 2.30.0) (34). The ANI of these contigs or *mcr*-flanking sequences was calculated using CD-HIT (version 4.8.1) (35) in three ways: (i) complete contigs with *mcr* genes with a 95% threshold, (ii) *mcr*-flanking sequences (3,000 bp upstream and 2,000 bp downstream) with a 95% threshold, and (iii) *mcr*-flanking sequences (3,000 bp upstream and 2,000 bp downstream) with an 80% threshold. Finally, we combined the three grouping results to obtain the final grouping result (see Table S1 in the supplemental material).

### Distance calculation for *mcr*-flanking sequences based on *k-*mers.

Distances of *mcr-*flanking sequences were estimated using Mash (version 2.3) (23) based on *k-*mers. The situation of *k-*mer sharing allowed equal comparisons of *mcr-*flanking sequences. The output was an edge list implying -E flag with the fields seq1, seq2, dist, p-val, and shared-hashes (Table S2). The dist field was the Mash distance, which ranged from 0 to 1, with 0 indicating identical sequences and 1 indicating dissimilar sequences. To make distance calculations efficient, a sketch length (-s) of 5,000 and a *k-*mer length (-k) of 13 were specified, while the other settings were the default settings. Thereafter, the output was converted into edge.csv as the input file for Gephi (version 0.9.2). A node information csv file, node.csv, was also created as the input file to establish an unoriented network graph for visualization. All isolates subjected to long-read sequencing were labeled in the graph.

### Isolate culturing, DNA extraction, and long-read sequencing.

Isolates were cultured for 12 to 16 h in LB broth (Land Bridge, Beijing, China) at 37°C. DNA was extracted using the Magen HiPure bacterial DNA kit, according to the manufacturer’s protocols (Magen, Guangdong, China). DNA purity and concentration were quantified using NanoDrop nucleic acid quantification (Thermo Fisher Scientific, Shanghai, China) and Qubit fluorometers (Thermo Fisher Scientific, Shanghai, China).

Sequencing libraries were prepared using the MinION Mk1C genomic DNA sequencing kit (Oxford Nanopore Technologies, Shanghai, China) with the rapid barcoding kit (catalog number SQK-LSK109) to barcode individual samples according to the manufacturer’s instructions. The MinION R9.4 flow cell (catalog number FLO-MIN106) was inserted into the MinION device, and the prepared library was loaded into the flow cell. MinKNOW v1.15.4 software was run to sequence the Salmonella genome for 72 h, including several core tasks: data acquisition, real-time analysis and feedback, base calling, data streaming, controlling the device, and ensuring that the platform chemistry was performing correctly to run the samples. Guppy v6.0.1 was used to call bases in real time and convert FAST5 files (Nanopore raw reads) to FASTQ format.

### Long-read assembly.

Unicycler (version 0.4.8) (24) was used to conduct a short-read-first hybrid assembly with long reads and Illumina reads obtained previously for each sample (our unpublished data) with default settings. Assembly statistics were generated using QUAST (version 5.0.2) (36) with default parameters. Outputs can be found in Table S3.

### Characterization of hybrid assemblies.

All 29 assemblies were scanned for the presence of antimicrobial resistance genes (ARGs) using ABRicate (version 1.0.1; T. Seemann [https://github.com/tseemann/abricate]) in the ResFinder database (29), with a 90% identification threshold and 60% minimum coverage. Contigs with *mcr* genes were selected and defined as plasmid (as previously defined) and chromosome. The obtained plasmids were classified based on the MOB and the mating pair formation (MPF) complex, and annotated ARGs by Plascad (37). The tree of the 27 obtained plasmids was constructed based on the distance of the plasmids, as described above.

### Short reads mapped to putative plasmids.

Short reads from 140 isolates were mapped (our unpublished data) to 27 putative plasmids, and align values were calculated. First, bowtie2-build from Bowtie 2 (version 2.4.4) (38) was used to index putative plasmids, and Bowtie 2 was used to map short reads to putative plasmids using 16 threads. Next, samtools view from samtools (version 1.14) (33) was used to convert the sam file to a bam file, and samtools sort was used to sort the bam file. samtools mpileup was used to call single nucleotide polymorphisms (SNPs) and indels. To ensure maximum sensitivity of plasmid detection, a base quality of 20, a mapping quality of 10, and a minimum of 20 high-quality mapped reads were all required. Taken together, we guaranteed that the align value of short reads for long sequences aligned to the assembly plasmid was higher than other values, usually >95%.

### A data set of complete plasmids carrying *mcr-1* or *mcr-3*.

Complete plasmids carrying the *mcr-1* or *mcr-3* gene were obtained from the NCBI database using esearch (version 16.2) (https://github.com/mpenet/esearch) on 19 January 2022. A query string with filters of plasmid, title of the complete sequence, and length from 2,000 to 500,000 bp was applied, while the remaining settings were the default settings. We then systematically curated the removal of repetitive plasmids included in both GenBank and the RefSeq release repository. The metadata accompanying each plasmid sequence were parsed from the associated GenBank files using extractmeta_plasmidDB.py (39). Finally, we collected 496 complete plasmids carrying the *mcr-1* gene and 31 carrying the *mcr-3* gene from the release repository. Appending our sequenced plasmids, the final data set included 518 *mcr-1*-positive and 36 *mcr-3*-positive complete plasmids. The accession numbers of *mcr-1*-positive and *mcr-3*-positive plasmid sequences and the accompanying metadata are available in Table S4. Also, the replicon types of *mcr-1*-positive and *mcr-3*-positive plasmid sequences and the accompanying metadata are available in Table S5.

### Replicon types and predicted mobility of plasmids.

Plasmids were predicted against replicon sequences and via mobility typing using MOB-typer from MOB-suite (version 2.0.0). MOB-typer predicts mobility based on MOB, MPF complex, and *oriT* genes. In short, a plasmid is putatively labeled as conjugative if it has both MOB and MPF, mobilizable if it has either MOB or *oriT* but no MPF, and nonmobilizable if it has no MOB and *oriT* (40).

### Calculation of distances of plasmids with *mcr* genes based on *k-*mers.

We estimated the distances between the *mcr*-flanking sequences using Mash (version 2.3), as previously described (23). This approach was also used for calculating pairs of plasmids applying similar flags. We also established an unoriented network for visualization using Gephi (version 0.9.2). All assembly plasmids were labeled in the graph. Distances of *mcr-1*-positive and *mcr-3*-positive plasmids are available in Table S6.

### Phylogenetic analysis and dissemination of isolates carrying *mcr-1* or *mcr-3*.

cgMLST loci of *mcr*-carrying Salmonella isolates were analyzed using SISTR_cmd to study their diversity and to understand the distribution of the different clones and plasmids carrying *mcr-1* or *mcr-3*. cgMLST loci were predicted by SISTR_cmd, and the different numbers between isolates were calculated by Python (version 3.9) to construct the phylogenetic tree based on 330 loci.

### Antimicrobial susceptibility testing.

The MICs of 15 commonly used antimicrobial agents were tested by broth dilution methods. The breakpoints of each agent were interpreted according to Clinical and Laboratory Standards Institute (CLSI) guidelines (41); E. coli ATCC 25922 was the quality control strain.

### Analysis of the genetic context of *mcr* genes.

The new *mcr*-flanking sequences, including 10,000 bp upstream of the *mcr* gene, the *mcr* gene, and 10,000 bp downstream, were extracted from hybrid assembly contigs carrying *mcr* genes. These flanking sequences were annotated using Prokka (version 1.14.6) (42) and BLAST (https://blast.ncbi.nlm.nih.gov/Blast.cgi), as mentioned above. GBK files from Prokka outputs were used as the input for visualization using the Easyfig (version 2.2.5) package (43).

### Data processing and visualization.

Data processing and visualization were performed using R (version 4.1.2), except for the network figure and the China map, which were made using Gephi (version 3.8.0) and Python (version 3.9.9) with the module pyecharts.

### Statistical analysis.

We used χ^2^ analysis to test the significance of differences in the proportions of *mcr*-positive isolates or replicon types between two time points, and Fisher’s exact test was used when the number of isolates was <10. Statistical significance (*P* value) was determined using R (version 4.1.2). A *P* value of less than 0·05 was considered statistically significant.

### Data availability.

The WGS data for 140 *mcr*-positive Salmonella isolates have been deposited in GenBank under BioProject accession number PRJNA813972.
