# Supplementary material for: Distribution and Transmission of Colistin Resistance Genes mcr-1 and mcr-3 among Nontyphoidal Salmonella Isolates in China from 2011 to 2020
Source: Microbiol Spectr. 2023 Jan 10;11(1):e03833-22. doi: 10.1128/spectrum.03833-22 (PMC9927481; doi:10.1128/spectrum.03833-22)
Supplement: Supplemental file 1 — Supplemental material. Download spectrum.03833-22-s0001.pdf, PDF file, 1.0 MB [file spectrum.03833-22-s0001.pdf]

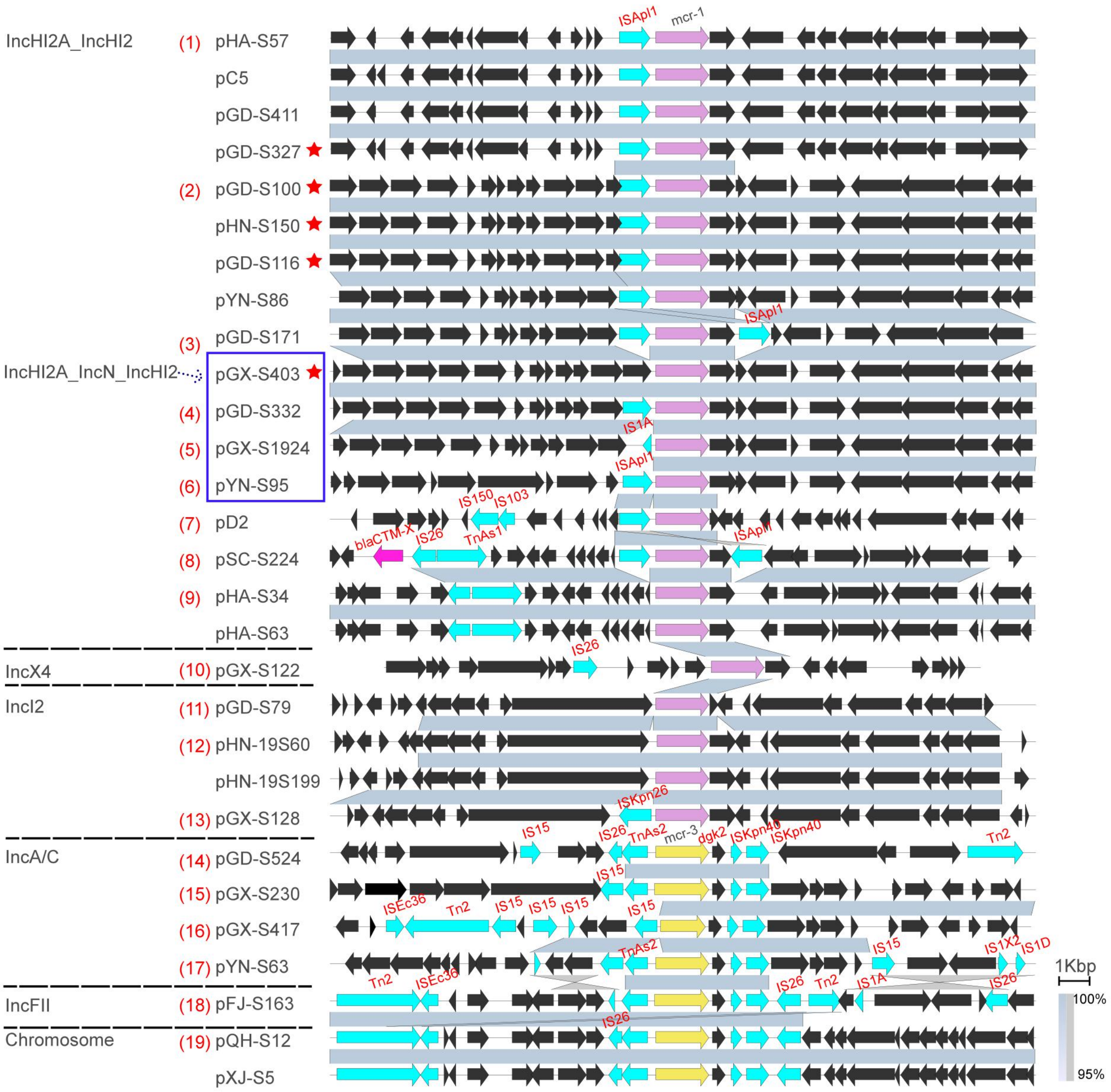

Figure S1. Genetic environments of *mcr-1* and *mcr-3* genes. The diverse genetic environments of *mcr-1* gene in 22 plasmids, *mcr-3* gene in 5 plasmids, and *mcr-3* gene in two chromosomes. Plum arrows indicate *mcr-1* gene, yellow arrows indicate *mcr-3* gene. Cyan arrows indicate insertion sequences or transposons, magenta arrows indicate ARGs, black arrows indicate other ORFs. Lightblue shading area between different sequences indicate the aligned regions. Red stars refer to plasmids predicted non-mobilizable by MOB_Suit.

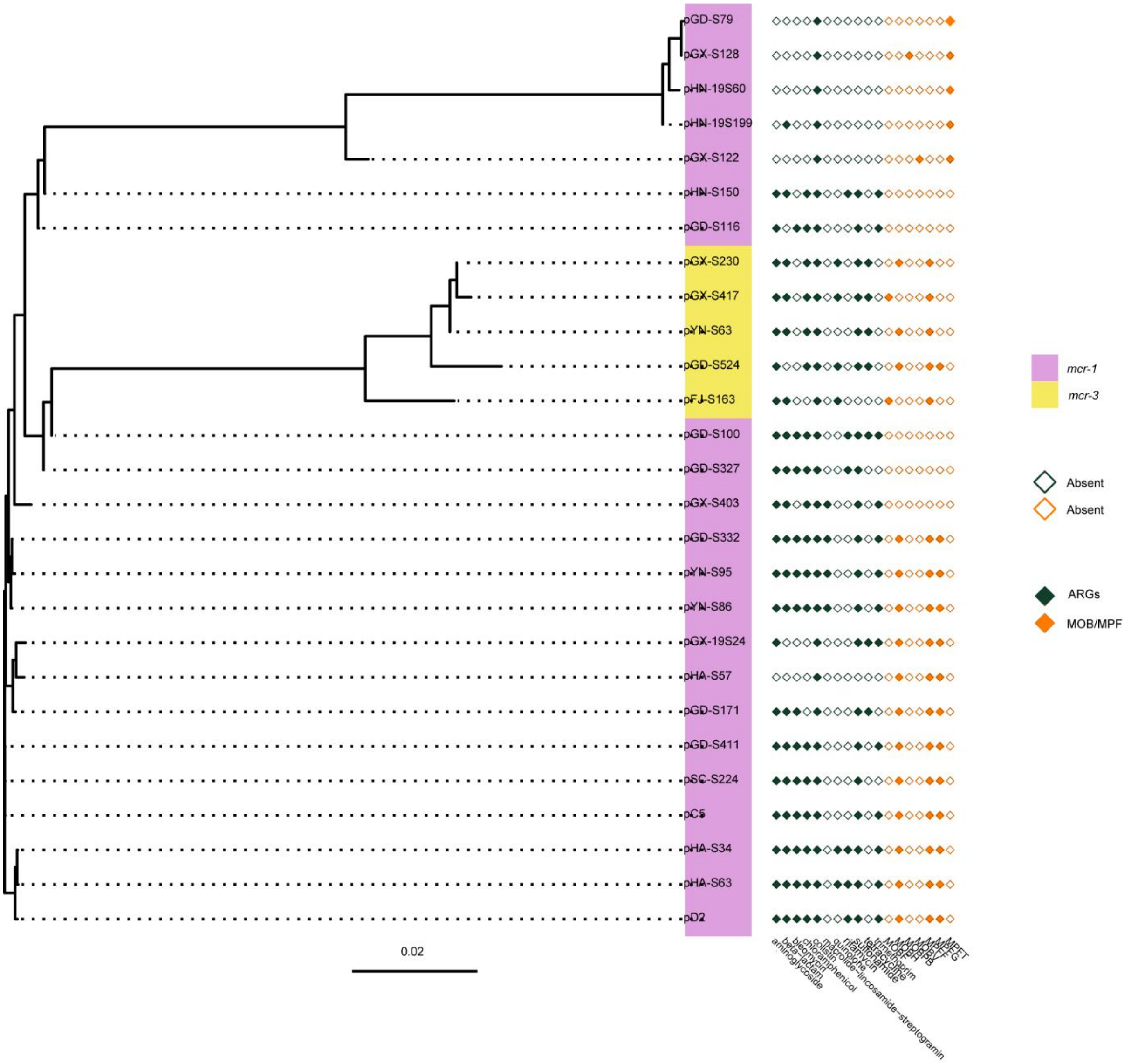

Figure S2. Phylogenetic tree of *mcr*-carrying plasmids in this study. Genetic relationships among *mcr*-carrying plasmids (n = 27). Phylogenetic tree construction is based on distance between pair of plasmids. The columns to the right of the tree indicate the pattern of aminoglycoside, ESBL, bleomycin, chloramphenicol, colistin, macrolide-lincosamide-streptogramin, quinolone, rifampicin, sulphonamide, tetracycline and trimethoprim, MOBF, MOBH, MOBPB, MOBV, MPFF, MPFG, and MPFT.

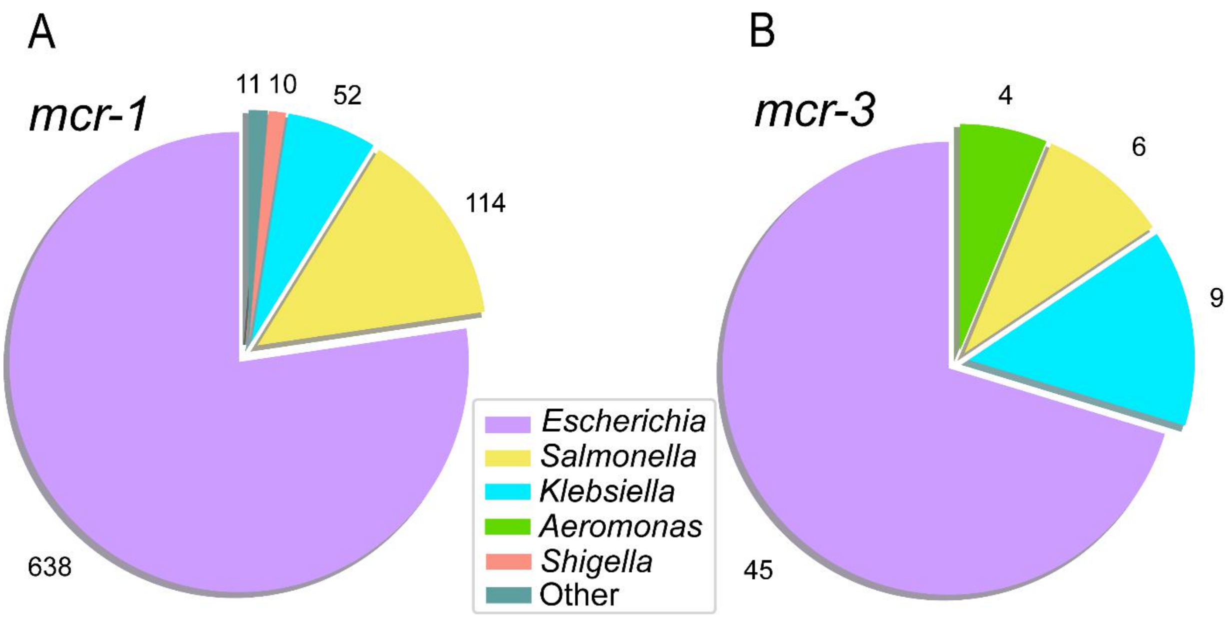

Figure S3. Diverse bacterial hosts of *mcr*-positive plasmids. (A) Diverse bacterial hosts of *mcr-1*-positive plasmids. (**B)** Diverse bacterial hosts of *mcr-3*-positive plasmids.

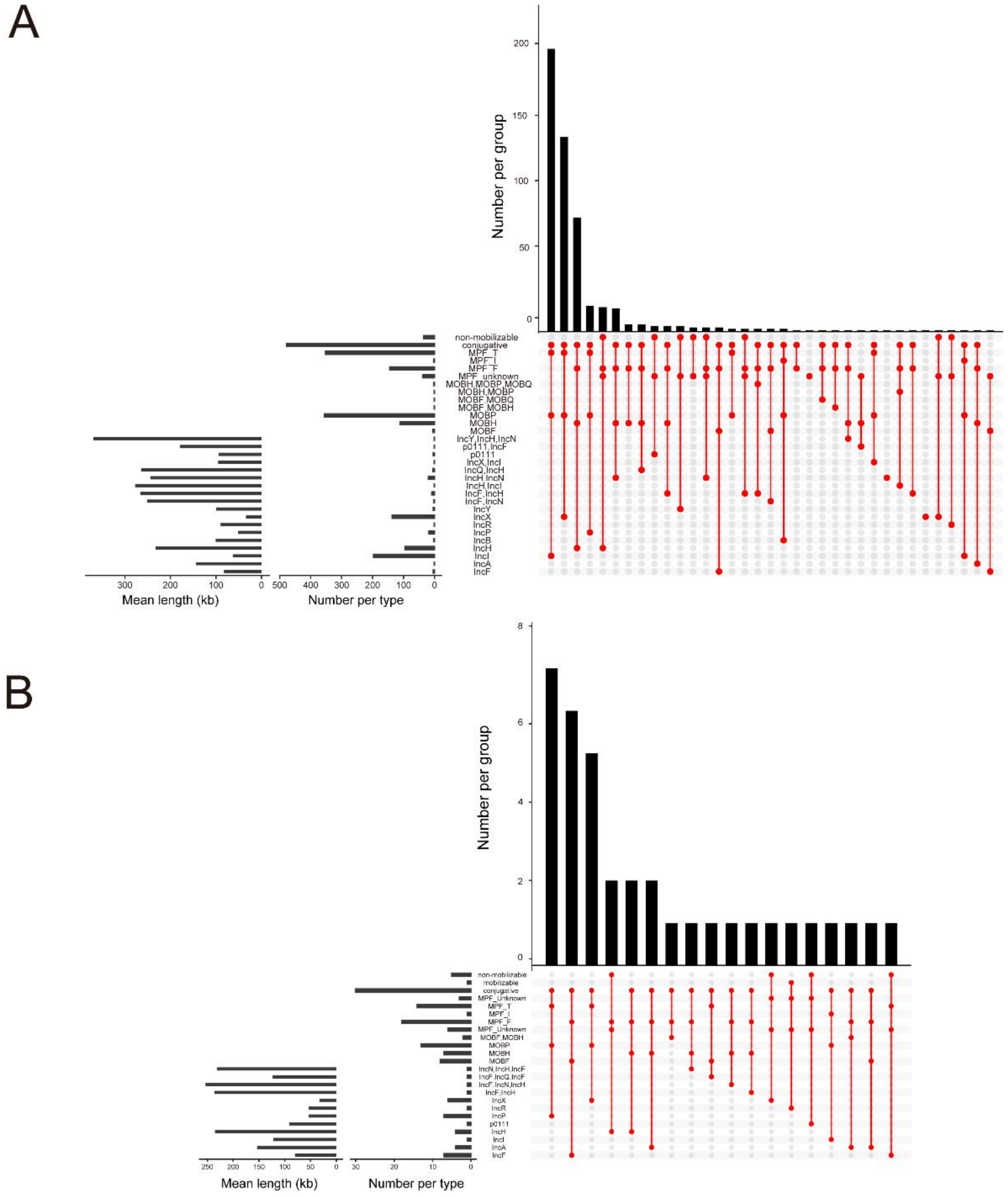

Figure S4. Traits of *mcr*-carrying plasmids. (A) Intersection plot of the combination of replicon types, MOB, MPF, and mobility found in the set of *mcr-1*-carrying plasmids (n = 518). **(B)** Intersection plot of the combination of replicon types, MOB, MPF, and mobility found in the set of *mcr-3*-carrying plasmids (n = 36).

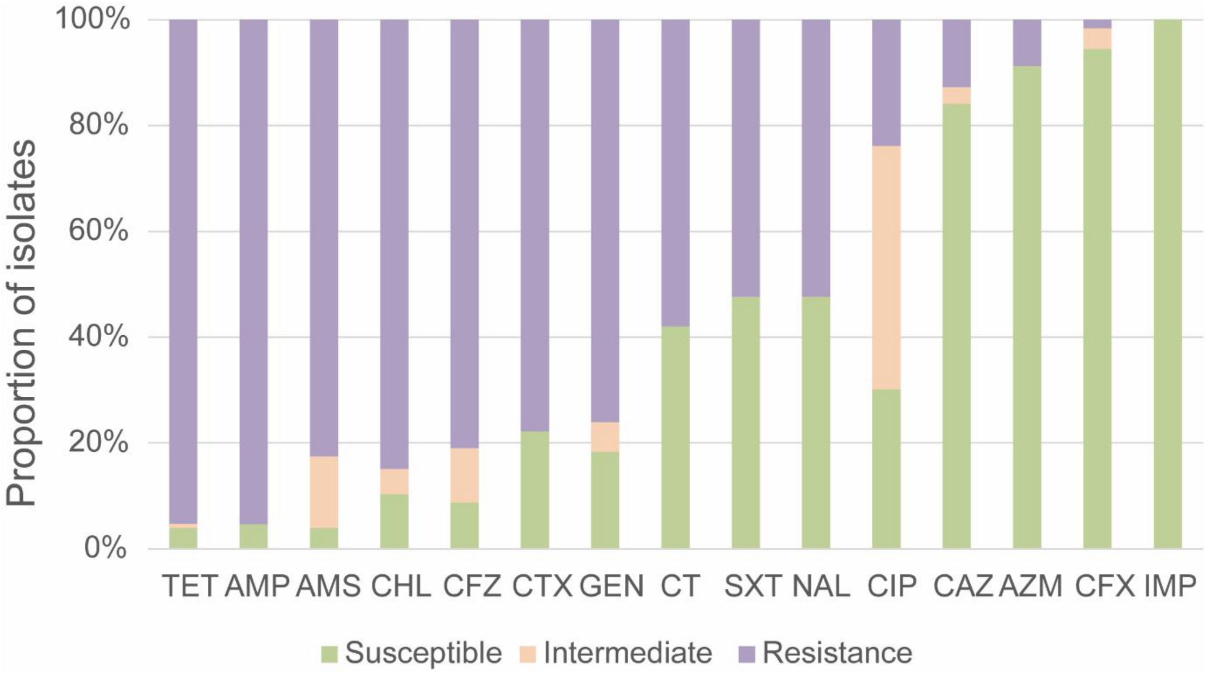

Figure S5. Antimicrobial resistance of *mcr*-positive *Salmonella.* Palegreen refer to susceptible rate, peachpuff refer to intermediate rate, and mediumpurple refer to antibiotic resistance rate. TET, tetracycline; AMP, ampicillin; AMS, ampicillin/sulbactam; CHL, chloramphenicol; CFZ, cefazolin; CTX, cefotaxime; GEN, gentamicin; CT, colistin; SXT, trimethoprim/sulfamethoxazole; NAL, nalidixic acid; CIP, ciprofloxacin; CAZ, ceftazidime; AZM, azithromycin; CFX, cefoxitin; IMP, imipenem.

Table S7. The province distribution of 7106 *Salmonella* isolates and 140 *mcr*-positive *Salmonella* isolates.

group	name	value	*mcr-1*	*mcr-3*
>500	Guangdong	1228	40	1
>500	Sichuan	723	8	1
>500	Guangxi	555	19	5
300~500	Hubei	451	1	0
300~500	Hunan	438	17	0
300~500	Jiangxi	422	8	0
300~500	Zhejiang	398	2	0
300~500	Anhui	375	1	0
100~300	Henan	275	7	0
100~300	Jiangsu	251	4	0
100~300	Yunnan	248	6	1
100~300	Beijing	226	1	0
100~300	Shaanxi	206	0	0
100~300	Jilin	192	5	0
100~300	Fujian	179	5	1
100~300	Tianjin	140	0	0
100~300	Heilongjiang	143	1	0
100~300	Shanghai	130	0	0
100~300	Shanxi	98	2	0
1~100	Shandong	88	0	0
1~100	Guizhou	72	0	1
1~100	Gansu	66	0	0
1~100	Xinjiang	52	0	1
1~100	Liaoning	38	0	0
1~100	Hebei	29	0	0
1~100	Qinghai	28	1	1
1~100	InnerMongolia	21	0	0
1~100	Ningxia	17	0	0
1~100	Chongqing	17	0	0
0	Aomen	0	0	0
0	Hainan	0	0	0
0	Taiwan	0	0	0
0	Xianggang	0	0	0
0	Tibet	0	0	0
0	The South China Sea Islands	0	0	0

Table S10. The proportion of *mcr*-positive isolates for 1,4,[5],12:i:-, Typhimurium, or total, respectively.

Date	No.	Serotypes	Proportion (%)	Prop.min	Prop.max
2011-15	93	total	4.3	0.18	8.42
2016	84	total	11.9	4.98	18.82
2017	652	total	11.35	8.92	13.78
2018	927	total	4.31	3.00	5.62
2019-20	1075	total	0.84	0.29	1.39
2011-15	37	*mcr*_Typhimurium	2.7	-2.52	7.92
2016	39	*mcr*_Typhimurium	7.69	-0.67	16.05
2017	240	*mcr*_Typhimurium	4.58	1.94	7.22
2018	384	*mcr*_Typhimurium	1.82	0.48	3.16
2019-20	409	*mcr*_Typhimurium	0.73	-0.10	1.56
2011-15	56	*mcr*_1,4,[5],12:i:-	5.36	-0.54	11.26
2016	45	*mcr*_1,4,[5],13:i:-	15.56	4.97	26.15
2017	412	*mcr*_1,4,[5],14:i:-	15.29	11.81	18.77
2018	543	*mcr*_1,4,[5],15:i:-	6.08	4.07	8.09
2019-20	666	*mcr*_1,4,[5],16:i:-	0.9	0.18	1.62

Table S11. The replicon types of *mcr-1/3*-carrying plasmids in 1,4,[5],12:i:- and Typhimurium.

Date	replicon type	No.	Proportion (%)	Total No.	Serotypes	Prop.min	Prop.max
2011-15	IncHI2A_IncHI2	3	3.23	37	Typhimurium	-0.36	6.82
2016	IncHI2A_IncHI2	9	10.71	39	Typhimurium	4.1	17.32
2017	IncHI2A_IncHI2	54	8.28	240	Typhimurium	6.16	10.4
2018	IncHI2A_IncHI2	30	3.24	384	Typhimurium	2.1	4.38
2019-20	IncHI2A_IncHI2	3	0.26	409	Typhimurium	-0.04	0.56
2011-15	IncX4	0	0	56	1,4,[5],12:i:-		
2016	IncX4	0	0	45	1,4,[5],13:i:-		
2017	IncX4	5	0.77	412	1,4,[5],14:i:-		
2018	IncX4	1	0.11	543	1,4,[5],15:i:-		
2019-20	IncX4	1	0.09	666	1,4,[5],16:i:-		
2011-15	IncI2	0	0				
2016	IncI2	0	0				
2017	IncI2	3	0.46				
2018	IncI2	1	0.11				
2019-20	IncI2	3	0.26				
2011-15	IncHI2A_IncN_IncHI2	1	1.08				
2016	IncHI2A_IncN_IncHI2	1	1.19				
2017	IncHI2A_IncN_IncHI2	3	0.46				
2018	IncHI2A_IncN_IncHI2	5	0.54				
2019-20	IncHI2A_IncN_IncHI2	2	0.18				
2011-15	IncFII	0	0				
2016	IncFII	0	0				
2017	IncFII	3	0.46				
2018	IncFII	0	0				
2019-20	IncFII	0	0				
2011-15	IncA/C2	0	0				
2016	IncA/C2	0	0				
2017	IncA/C2	4	0.61				
2018	IncA/C2	3	0.32				
2019-20	IncA/C2	0	0				
